# Impact of tumor markers on diagnosis, treatment and prognosis in CNS germ cell tumors: correlations with clinical practice and histopathology

**DOI:** 10.1007/s10014-023-00460-x

**Published:** 2023-03-30

**Authors:** Hirokazu Takami, Christopher S. Graffeo, Avital Perry, Caterina Giannini, Yoichi Nakazato, Nobuhito Saito, Masao Matsutani, Ryo Nishikawa, David J. Daniels, Koichi Ichimura

**Affiliations:** 1 Department of Neurosurgery, The University of Tokyo Hospital, 7-3-1 Hongo, Bunkyo-ku, Tokyo, 113-8655 Japan; 2 Department of Neurologic Surgery, Mayo Clinic, Rochester, MN USA; 3 Department of Laboratory Medicine and Pathology, Mayo Clinic, Rochester, MN USA; 4 Department of Pathology, Hidaka Hospital, Gunma, Japan; 5 Division of Pediatric Neuro-Oncology, Saitama Medical University International Medical Center, Saitama, Japan; 6 Department of Brain Disease Translational Research, Juntendo University Graduate School of Medicine, Tokyo, Japan

**Keywords:** Germ cell tumor, Germinoma, Tumor marker, Histopathology, Histology

## Abstract

Tumor markers in CNS germ cell tumors (GCTs) include human chorionic gonadotropin (HCG) and alpha fetoprotein (AFP), which have significant diagnostic implications, as elevation of either one leads to clinical diagnosis of non-germinomatous GCTs without histopathological confirmation, justifying intensified chemotherapy and irradiation. The current study, based on an international cohort of histopathologically verified GCTs that underwent biopsy (*n* = 85) or resection (*n* = 76), sought to better define the clinical role and prognostic significance of tumor markers from serum and CSF in this challenging patient population. We found that HCG was elevated only in cases with a germinoma or choriocarcinoma component, and there existed a clear cut-off HCG value between the two. AFP was often elevated in GCTs without a yolk sac tumor component, especially immature teratoma. HCG was elevated only in CSF in 3-of-52 cases, and AFP was elevated only in serum in 7-of-49 cases, emphasizing the potential utilization of both serum and CSF studies. Immature teratoma demonstrated unfavorable prognosis independent of tumor marker status, with 56% 5-year overall survival; however, co-existent germinoma components indicated a more favorable prognosis. Taken together, the study findings emphasize the importance for routine assessment and guarded interpretation of tumor markers in CNS GCTs.

## Introduction

Central nervous system (CNS) germ cell tumors (GCTs) are a family of predominantly malignant neoplasms impacting pediatric, adolescent and young adult populations, with a median age at diagnosis from 12 to 16 years old [1]. Overall, GCT patients are overwhelmingly male, with >90% of pineal lesions [2], and approximately 50% of neurohypophyseal lesions arising in men [3]. GCTs arise primarily in midline structures including the pineal gland and neurohypophysis most frequently, followed by basal ganglia, thalamus, cerebrum, cerebellum, and spinal cord [4, 5].

Histopathologically, germinoma accounts for 50–60% of GCTs, while non-germinomatous GCTs (NGGCTs) are a minority [1]. According to WHO classification, NGGCTs include mature and immature teratoma (MT and ImT), teratoma with somatic-type malignancy, yolk sac tumor (YST), choriocarcinoma (CC), embryonal carcinoma (EC), and mixed GCT, which may demonstrate any combination of the aforementioned histological components [6]. Germinoma responds well to platinum-based chemotherapy and whole ventricular irradiation (WVI), with > 90% survival in long-term follow-up studies [1, 7, 8]. By contrast, NGGCTs are prognostically unfavorable in comparison and demonstrate only 60–70% long-term survival, in particular among tumors with a malignant component (YST, CC, and EC) [1]. Correspondingly, treatment protocols are more aggressive and frequently incorporate intensified chemotherapy regiments with both platinum and alkylating agent chemotherapy as well as well as irradiation, although radiation coverage protocols are heterogeneous and vary by treatment location and active clinical trial participation, among other factors [9–12].

Teratoma biology is heterogeneous, and although the MT phenotype is less aggressive and treatment is oriented around surgical resection alone, ImT treatment remains an area of active study. At present, COG clinical trials include ImT with other NGGCTs if tumor markers is elevated or the diagnosis has been histopathologically proven; SIOP protocols allow for ImT treatment planning on a case-by-case basis [8, 13, 14]. In Japan, ImT is classified within the “intermediate risk group,” prompting treatment with chemotherapy and irradiation that is more intensive than germinoma protocols but less aggressive than NGGCTs with a dominant malignant component [5]. Correspondingly, diagnostic differentiation between germinoma, MT/ImT, and phenotypically malignant NGGCTs has significant implications with regard to treatment pathways, patient education, and prognostic counseling.

GCT diagnosis is established through various methods, which may incorporate imaging findings, histopathological specimens, and tumor markers from serum or cerebrospinal fluid (CSF). In COG and SIOP protocols, tumor marker thresholds are defined for the diagnosis of NGGCTs (with a malignant component), which in turn prompts protocol-based therapeutic assignment [11]. However, a preceding report argued that elevation of tumor markers was not limited to NGGCTs with a malignant component [15], while another study demonstrated that HCG RNA was expressed across all histological subtypes [16]. Correspondingly, establishing a GCT diagnosis and proceeding with intensive therapies on the basis of tumor markers alone remains controversial and potentially risky. In tandem, histopathological diagnosis based on a small biopsy specimen predisposes to sampling error and associated under-estimation in the prevalence of malignant components. Collectively, these complexities have yielded a highly heterogenous set of protocols with regard to how tumor markers are incorporated into algorithms for GCT diagnosis and treatment across the world [17].

The goal of the current study was to assess the distribution of elevated tumor markers in resection-based histopathologically confirmed GCT cases, as well as the relationship between tumor markers and histopathologic diagnosis, with particular attention to the range of marker abnormalities observed in GCTs without a YST or CC component. Further emphasis was placed on examining the relative yield of tumor markers measured from serum, CSF, or both, as well as the relationship between marker levels, clinical phenotype, and treatment response in ImT.

## Methods

Retrospective cohort study of consecutive, neurosurgically managed, intracranial GCTs treated at Mayo Clinic during the study period, 1988–2017. Initial query yielded 80 primary cases (non-metastatic; non-recurrent). All histopathological diagnoses were confirmed by a board-certified neuropathologist on staff at Mayo Clinic. All pertinent aspects of the current study were approved and overseen by our institutional review board, including a consent waiver for a minimal-risk study.

The intracranial GCT Genome Analysis Consortium (iGCT Consortium) database was queried, and 154 primary intracranial GCTs were identified and included in the study. Central histopathological re-review according to the contemporary WHO classification of tumors of the central nervous system was performed by a single expert neuropathologist (YN). This component of the investigation was approved by the ethics committee of the National Cancer Center, Tokyo, Japan, and local institutional review boards.

In this subgroup, all patients with histopathologic diagnosis and baseline tumor markers were included. Eligible tumor marker results included those measured in serum or CSF, collected either the preoperatively or intraoperatively. The combined study cohort correspondingly included 161 cases: 40 from Mayo Clinic, 121 from iGCT consortium. One case was omitted from the preceding study [15], as the timing of tumor marker measurement and surgical resection was not strictly identical.

Overall, 76 cases were diagnosed using resection specimens, while 86 specimen were obtained by biopsy only. Both HCG and AFP were obtained in 86 cases; only one marker was reported in the remainder. For patients with both serum and CSF tumor markers, the higher result was used for study analysis. Thresholds for positive tumor marker were defined using COG clinical trial protocols: 100IU/L for HCG and 10 ng/mL for AFP [13, 14].

## Results

### Distribution of elevated tumor markers by histopathologic diagnosis

**Fig. 1:**
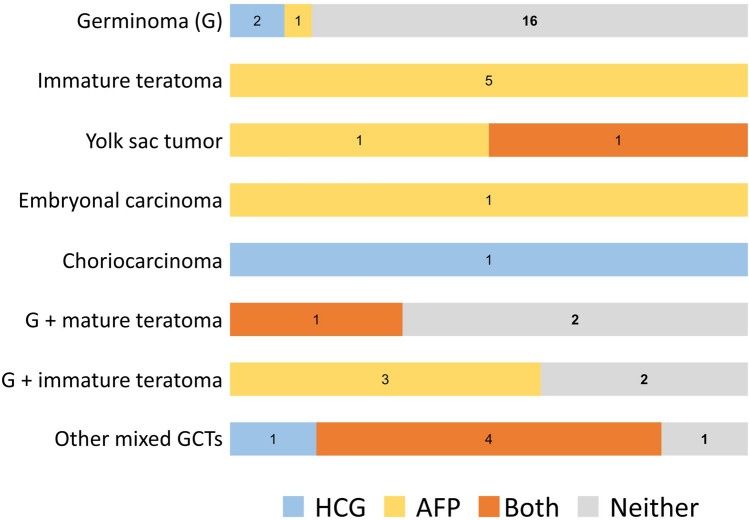
Distribution of number of cases with elevated tumor markers (HCG, AFP, both, or neither) per confirmed histopathology diagnosed on resected tumor sample. All immature teratoma cases, either with or without a germinoma component, showed elevated AFP

Distribution of marker-positive and -negative cases depending on histopathology, among cases where the samples were resection-based and both tumor markers were measured, is shown in Fig. 1. Most germinoma cases (16-of-19; 84%) did not show elevation in either tumor marker. All six ImT cases and 3-of-5 ImT cases with a germinoma component (60%) showed elevated AFP. Both YST cases demonstrated elevation in AFP, one of which was also notable for elevated HCG as well; one choriocarcinoma demonstrated elevated HCG.

### HCG in GCTs other than germinoma and choriocarcinoma; and AFP in GCTs other than yolk sac tumor

**Fig. 2:**
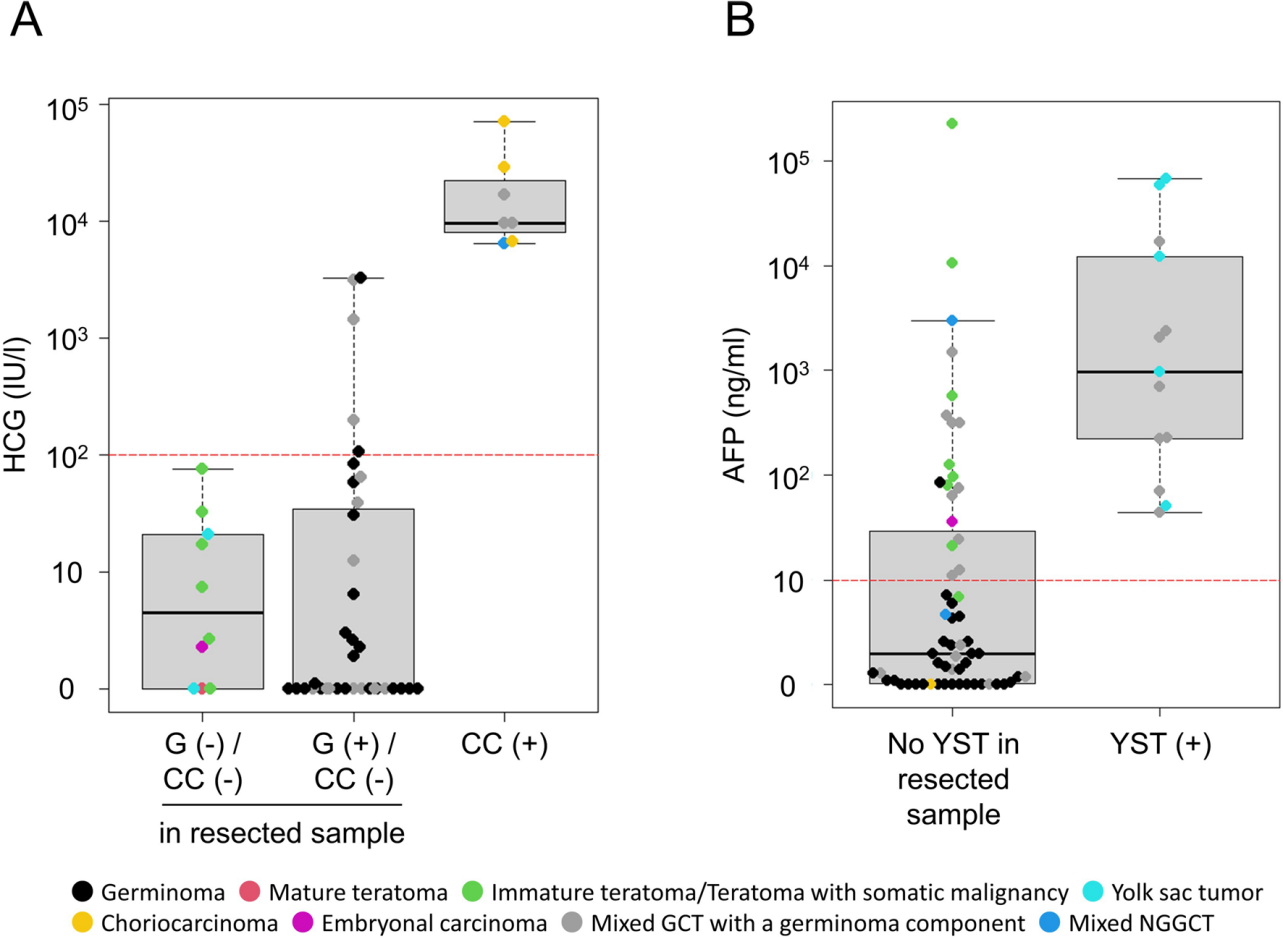
**A** While all the cases with a choriocarcinoma (CC) component showed extremely high HCG levels, cases with a germinoma (G) component without a CC component confirmed on resected samples also showed higher HCG level above the threshold of 100 IU/L. **B** All the cases with a yolk sac tumor (YST) component confirmed either in biopsy or resection samples showed high AFP level above the threshold of 10 ng/mL. Those cases without a YST component confirmed on resected samples also showed elevated AFP levels, mostly contributed by cases with a teratoma component

All cases with a choriocarcinoma component showed marked HCG elevation, while many cases with a germinoma component demonstrated HCG elevation of at least 100 IU/L—an abnormality that was exclusively observed in cases with either germinoma or CC components. The highest HCG value in cases without a CC component was 3267.5 IU/L in CSF; the lowest HCG value in cases with a CC component was 6390 IU/L in serum (Fig. 2A). All cases with a histology proven YST component from either biopsy or resection demonstrated AFP > 10 ng/mL; however, many cases without a confirmed YST component also demonstrated AFP elevation, and the ranges of abnormal AFP in these groups overlapped (Fig. 2B).

### Comparison of tumor markers between serum and cerebrospinal fluid

Fifty-two cases reported HCG in both serum and CSF. Among these, 49 showed concordant results, while three had a mismatch with HCG elevation seen in CSF only. Histopathology in these three cases was germinoma, germinoma with MT, and germinoma with both ImT and YST.

**Fig. 3:**
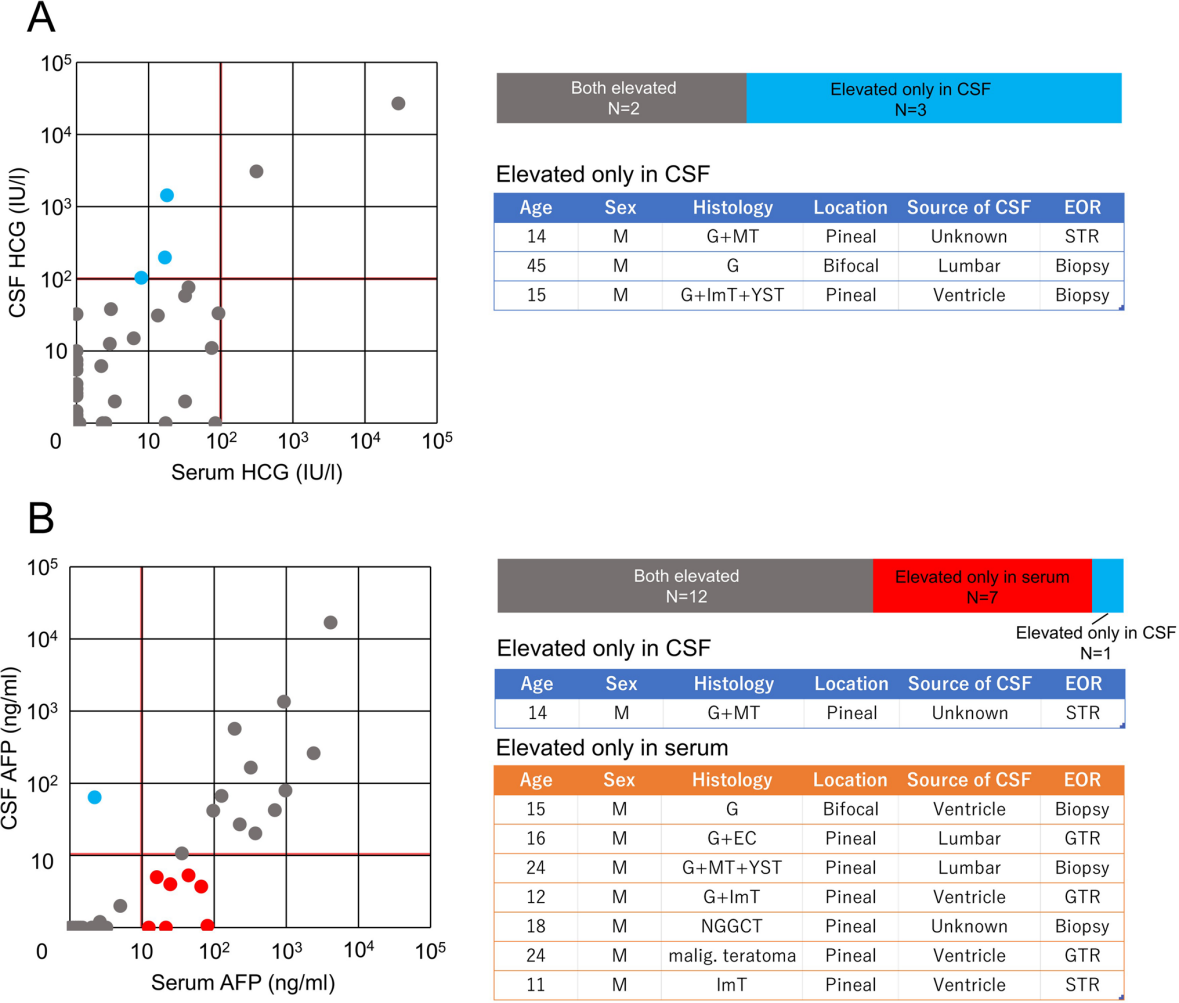
**A** Comparison of HCG level in blood serum and cerebrospinal fluid (CSF) indicated that 3 out of 52 cases where both were checked showed a discordance in terms of elevation above 100 IU/L. All of these cases showed elevation only in CSF, harbored a germinoma component, and lesions were located at pineal gland. **B** Comparison of AFP level in blood serum and CSF indicated that 7 out of 49 cases where both were checked showed a discordance in terms of elevation above 10 ng/mL. Almost all of these cases showed elevation only in serum, and lesions were located at pineal gland

Forty-nine cases reported AFP in both serum and CSF. Among these, 41 showed concordance, while eight had a mismatch, with serum AFP elevated in seven cases and CSF AFP elevated in 1. The only case that demonstrated isolated CSF AFP elevation was germinoma with MT. Histopathology for the seven cases with isolated serum AFP elevation were germinoma, germinoma with EC, germinoma with MT and YST, germinoma with ImT, NGGCT not otherwise specified, teratoma with somatic-type malignancy, and ImT. In all of the mismatch cases either in HCG or AFP, tumor was located at pineal gland with (*n* = 2) or without (*n* = 8) neurohypophysis (one case was an overlap in HCG and AFP) (Fig. 3A, B).

### Tumor marker elevation in immature teratoma and prognostic correlation

**Fig. 4:**
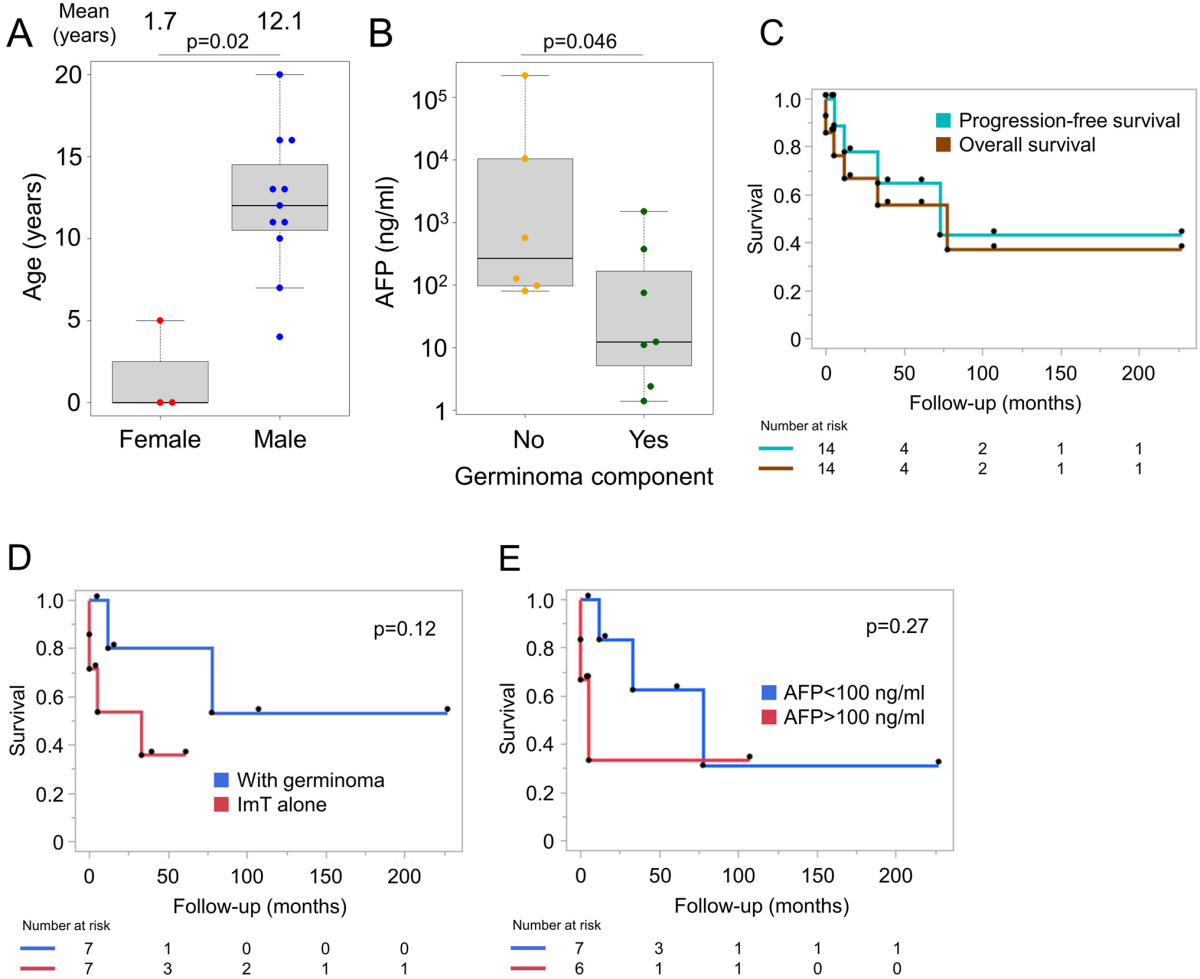
**A** Female immature teratoma (ImT) cases were significantly younger than male ImT cases (*p* = 0.02). **B** Cases with a germinoma component showed lower AFP level than “pure” ImT cases (*p* = 0.046). **C** Kaplan–Meier curves of progression-free and overall survival (PFS, OS) were shown. Five-year PFS and OS were 64.8% and 55.6%, respectively. **D** Cases with a germinoma component showed a better OS than those without, although statistically marginal (*p* = 0.12). **E** There was a trend of worse OS in cases with AFP>100 ng/mL than those AFP<100 ng/mL, but the difference was not statistically significant (*p* = 0.27)

All ImT demonstrated AFP elevation, and among cases with resection-based histopathology, ImT was diagnosed in 7, or present mixed with germinoma in 7. Among these 14 patients, there were three female patients, and the mean age at diagnosis was significantly younger in that group (1.7 vs 12.1 years, *p* = 0.02, Fig. 4A). AFP in serum or CSF ranged from 12.4 to 224865 ng/mL, with a median value of 97 ng/mL. Cases with a germinoma component showed lower AFP level (*p* = 0.046, Fig. 4B). Except for two cases that progressed rapidly after resection and prior to initiation of adjuvant therapies, all ImT received chemotherapy and irradiation, either via whole-brain (*n* = 4), ventricular (*n* = 6) or focal strategies (*n* = 2). Five-year progression-free and overall survivals were 64.8% and 55.6%, respectively (Fig. 4C). Cases with a germinoma component had favorable prognoses as compared to isolated ImT, although the difference in overall survival was not statistically significant (*p* = 0.12, Fig. 4D). A similar trend was observed between shorter survival and AFP >100 ng/mL; however, the difference between the groups was not statistically significant (*p* = 0.27, Fig. 4E). Key details regarding the ImT cases included in the current study are summarized in Table 1.

**Table 1:** Summary of 14 cases of immature teratoma with or without germinoma component and mature teratoma component, histopathologically diagnosed on resected specimen

ID	Age	Sex	Histopathological Dx	Location of tumor	Chemotherapy	EOR	Radiation	F/U (days)	Recurrence/progression	F/U (days)	Outcome	Serum HCG (IU/L)	CSF HCG (IU/L)	Serum AFP (ng/mL)	CSF AFP (ng/mL)	Source of CSF
1	0	F	ImT	Frontal lobe	No	PR	Not performed	4	No recurrence	4	Dead	2.7		224,865		ND
2	16	M	ImT	Pineal	PE	GTR	WB (30Gy) + focal (20 Gy)	162	Recurrence	162	Dead	0.1	32.4	192.2	568.59	V
3	10	M	ImT	Pineal	PE	GTR	WB (30Gy) + focal (20 Gy)	1862	No recurrence	1862	Alive	0.1	7.49	97	41.76	V
4	11	M	ImT	Pineal	PE	STR	WB (30Gy) + focal (20 Gy)	1011	Progression	1011	Dead	0.1	0.1	80.7	1.06	V
5	0	F	ImT	Ventricle	No	STR	Not performed	3	No recurrence	3	Dead			10,481		
6	13	M	ImT	Basal ganglia	PE	GTR	WB (23.4Gy) + focal (27 Gy)	123	No recurrence	123	Alive			126.2	66.9	V
7	4	M	ImT	Pineal	PE	GTR	WV (23.4Gy) + focal (37.8 Gy)	1204	No recurrence	1204	Alive	17.2	0.1			
8	12	M	G + ImT	Pineal	PE	GTR	Focal (ND)	6909	No recurrence	6909	Alive	0.1	0.1	12.4	0.1	V
9	7	M	G + ImT	Pineal	PE	GTR	Focal (54 Gy)	2219	Recurrence	2363	Dead	0.1	0.1	1.4	0.1	V
10	5	F	G + ImT	Neurohypophysis	PE	STR	WV (ND) + focal (50)	3266	No recurrence	3266	Alive			1492.5		
11	16	M	G + ImT	Pineal	CARE	STR	WV (ND)	365	Recurrence	365	Dead	64		74.5		
12	20	M	G + ImT	Pineal	ICE	GTR	WV (24 Gy)	476	No recurrence	476	Alive	0.1	0.1	2.4	0.1	V
13	11	M	G + ImT	Pineal	PE	GTR	WV (23.4 Gy) + focal (27 Gy)	150	No recurrence	150	Alive	0.1	0.1	372.8	20.2	V
14	13	M	G + ImT	Pineal	ICE	GTR	WV (ND)	149	No recurrence	149	Alive			11		

*F* female; *M* male; *G* germinoma; *ImT* immature teratoma; *PE* cisplatin + etoposide; *CARE* carboplatin + etoposide; *ICE* ifosfamide + cisplatin + etoposide; *EOR* extent of resection; *PR* partial resection; *STR* subtotal resection; *GTR* gross total resection; *WB* whole brain; *WV* whole ventricle; *F/U* follow-up; *V* ventricle; *ND* no data

## Discussion

The current study extends our preceding work regarding the evolving understanding of tumor markers in CNS GCTs [15]. Our previous study demonstrated that elevation of tumor marker alone cannot exclude the possibility of germinoma or teratoma within the true histopathologic profile of a given GCT. These new results add further depth to this understanding by highlighting the wide and nuanced distribution of both positive and negative tumor markers across the range of histopathological entities in resection-based diagnoses. Further, our findings reinforce the need of examining tumor markers both in serum and CSF, and provide new data regarding the relationship between prognosis and tumor markers in the setting of ImT following protocol-based adjuvant chemoradiation.

Unsurprisingly, some of the pure germinoma cases (e.g., without any NGGCT) demonstrated CSF HCG elevations as high as 3267.5 IU/L; however, we identified an important HCG threshold (3300–6300 IU/L) beyond which a CC component is almost guaranteed (Fig. 2A). Elevated AFP was observed in all cases with a YST component, but many of the cases without YST demonstrated similar AFP elevations in a range that was comparable to the YST tumors themselves. Out of 18 cases with elevated AFP and without a YST component, two cases had a MT component, 12 cases had an ImT component and one case was teratoma with somatic-type malignancy; 83% at least one teratoma component (Fig. 2B).

GCT protocols have long assumed that serum and CSF tumor markers are highly concordant; however, measurement of both has remained the current standard-of-care [18]. The current study revealed that, while serum and CSF tumor markers are typically concordant, outliers are not uncommon, and key information is expected from both values, in various circumstances. More specifically, CSF screening was more sensitive in examining in detecting HCG abnormalities, while serum was more sensitive to AFP elevations, indicating that although additional study may refine testing guidelines, for the foreseeable future collection of both tumor markers from CSF and serum is recommended to be continued as the GCT standard-of-care [8, 11, 13, 14, 19]. Of particular interest, instances of CSF-serum mismatch for a single tumor marker all occurred in patients with pineal GCTs, a minority of which also had a neurohypophyseal lesion. This may reflect underlying systematic patterns, such as the increased prevalence of both marker abnormalities among NGGCTs, and NGGCTs in the pineal region; however, underlying differences in GCT biology between these sites have not been fully explored, and may more fully explain these phenotypic trends.

ImT remains perhaps the greatest challenge in clinical GCT management, with limited understanding of clinical phenotypes, optimal treatment, and individualized prognosis at present. Those with elevated markers are treated in accordance with COG and SIOP protocols for NGGCTs, while ImT classified as intermediate-risk in Japan undergo a modified intensity treatment protocol that approximates a midpoint between standard germinoma care and high-intensity protocols for NGGCTs with a malignant component. In the current study, ImT cases demonstrated unfavorable prognoses overall, a finding that was more pronounced among those ImT lacking a germinoma component. OS in the entire ImT cohort was 56% at 5 years (Fig. 4C). Unexpectedly, AFP level did not appear significantly associated with prognosis (Fig. 4E). A sensitivity analysis built around the subtyping of ImT presenting at less than 6 years of age (type I) as compared to other CNS GCTs [20] showed no difference in prognosis at time-of-diagnosis or survival outcomes when the study cohort was dichotomized across the same age threshold (*n* = 4 vs. 10; data not shown). Overall, although interpretation of our study data are restricted by the relatively small sample size, the profound aggressiveness noted in the ImT subgroup emphasizes the need for intensive multimodality treatment incorporating surgery, chemotherapy, and irradiation. Of particular note, radiation field targeting for ImT remains an area of marked clinical heterogeneity, and an important area for future research, ideally in a randomized setting.

### Limitations

The current study is subject to a wide range of limitations, including those pertinent to essentially all observational research, such as confounding, selection bias, and incomplete data capture. Similarly, given the rarity of GCT diagnoses in general, the study sample is relatively small, in spite of being built on of two robust and prominent international registries. To that same end, as the study database was generated by incorporating case data from multiple institutions in Japan and the United States, management practices and documentation practices within the cohort are inevitably heterogeneous. Among the meaningful consequences of these differences are the lack of consistency with regard to what tumor markers were captured for which patients, resulting in further restriction of the sample sizes for various subgroup analyses. Notwithstanding, our work represents one of the largest, best-followed, and most robustly characterized cohorts of GCTs in the neurosurgical literature, which we anticipate will helpfully inform future guidelines and research efforts.

## Conclusion

The definitive clinical role of tumor markers in CNS GCT diagnosis and treatment remains incompletely understood. HCG elevation of greater than 3300–6300 IU/L is associated with NGGCT with a choricarcinoma component and elevation of AFP is noted in both YST and non-YST GCTs. CSF is more sensitive for HCG, while serum is more sensitive for AFP, emphasizing the need to obtain both samples and markers. ImT prognosis is unfavorable, independent of AFP elevation, highlighting the need for more sophisticated clinical trials dedicated to optimizing treatment protocols for these uncommon and highly challenging tumors.

## Data Availability

The data that support the findings of this study are available on request from the corresponding author, as long as it does not compromise the privacy of the patients included in this study.
